# Isotomidae of Japan and Asiatic part of Russia. II. The genus *Tetracanthella* of the Far East

**DOI:** 10.3897/zookeys.855.33000

**Published:** 2019-06-13

**Authors:** Mikhail Potapov, Alexey Brinev, Xin Sun

**Affiliations:** 1 Moscow State Pedagogical University, Kibalchich str., 6, korp. 3, Moscow 129278, Russia Moscow State Pedagogical University Moscow Russia; 2 J.F. Blumenbach Institute of Zoology and Anthropology, University of Göttingen,37073 Göttingen, Germany University of Göttingen Göttingen Germany; 3 Key laboratory of Wetland Ecology and Environment, Northeast Institute of Geography and Agroecology, Chinese Academy of Sciences, Changchun 130118, China Northeast Institute of Geography and Agroecology, Chinese Academy of Sciences Changchun China

**Keywords:** α-taxonomy, Collembola, the Far East of Russia, Japan

## Abstract

The paper considers new and little-known species of the genus *Tetracanthella* distributed in the Far East of Russia and in Japan. Sensillar chaetotaxy and labial palp, two less known morphological characters for the genus, are discussed. Two new species *T.annulata***sp. nov.** and *T.tardoki***sp. nov.** are described; *T.manschurica* Kutyreva, 1980 and *T czernovae* Kutyreva, 1980 are redescribed. For the latter species a lectotype and paralectotypes are designated. Remarks are provided for *T.sylvatica* Yosii, 1939. A second undescribed species is recorded for Japan. New records for *T.orientalis* Martynova, 1977 and *T.sibirica* Deharveng, 1987 are listed.

## Introduction

*Tetracanthella* is a typically Holarctic genus and is one of largest in the family (Deharveng 1987). The maximal diversity is located in Europe where 80 species are known. The Asiatic fauna is less understood but is obviously not so rich. So far only 17 species are recorded in Asia. Our study is a result of examination of large collections coming from the Far East of Russia. In the area under study, the species of the genus is a rather rare and unpredictable component of Collembolan communities. Ecological niche of the genus is more limited here than in Europe: corticolous species absent, high mountain forms are very rare (*T.tardoki* sp. nov.). Most east-Asiatic species of *Tetracanthella* are damp litter dwellers. Taxonomically, the species belong to Asiatic or American groups (‘*sylvatica*’, ‘*stebaevae*’, and ‘*ethelae*’ groups). Few species (*T.martynovae* and *T.sibirica*) occurs only in the arctic zone of the Far East and belong to generally European ‘*wahlgreni*’ group. We list below all the species of the region, redescribe little-known species, and describe two new ones. This paper is our second special contribution to taxonomy of Asiatic species of Isotomidae of Russia and Japan (Potapov et al. 2018). Following our results, the fauna of *Tetracanthella* of the Far East of Russia and Japan is represented by eight and two species, respectively, including still undescribed forms.

**Abbreviation used**

**A, B, C, D, E** papillae of labial palp following notation of Fjellberg (1999)

**A.B.** A. Brinev

**A.F.** A. Fjellberg

**A.G** A. Geras’kina

**A.K.** A. Kuprin

**a1** medial mesochaetae on Abd.V

**a2** medial macrochaetae on Abd.V

**Abd.** abdominal segments

**Alt** altitude

**Ant.** antennal segments

**Ap** unpaired chaetae in anterior part of head

**B5, X** chaetae on tibiotarsus 3 following notation of Deharveng (1983)

**dA, dH** diameter of ocellus A and H

**eAS** external pair of anal spines

**M.P.** M. Potapov

**Md, Mdl, Ml** macrochaetae in dorsal, dorso-lateral and lateral position

**ms** micro s-chaeta(e) or ms-chaeta(e)

**MSPU** Moscow State Pedagogical University

**PAO** postantennal organ

**N.K.** N. Kuznetsova

**p1, p3** chaetae of p-row on tergites

**PAO** postantennal organ

**p3** chaetae of p-row on head following notation of Deharveng (1987)

**pp** chaetae of pp-row on head following notation of Deharveng (1987)

**s** in the text and figures, macro s-chaeta(e) or s-chaeta(e)

**s**’ male s-chaeta on Ant.3 in lateral position

**Th.** thoracic segments.

### Towards the knowledge of the taxonomic characters regarding the species of the Far East of Russia

**Figures 1–4.**
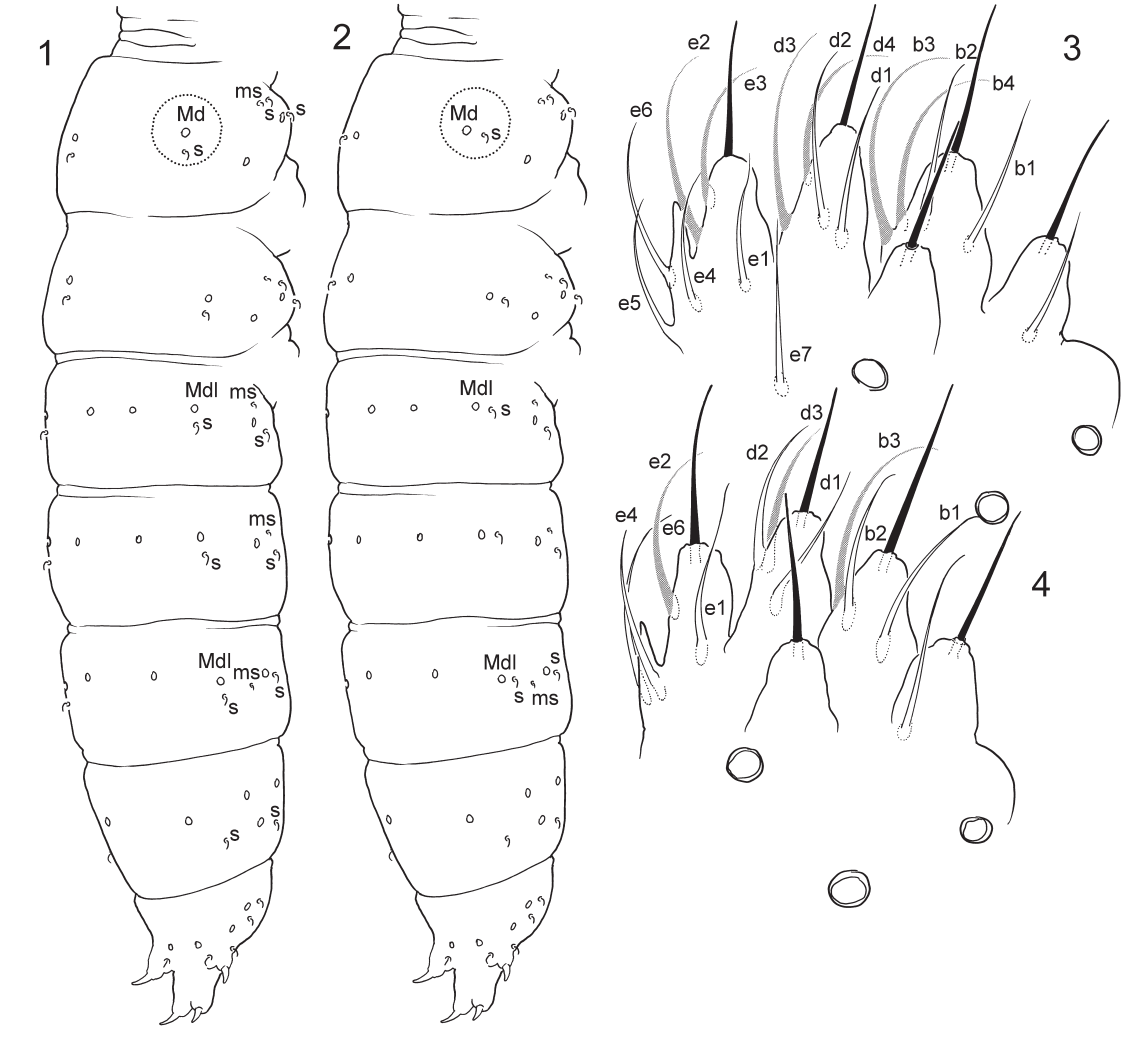
*Tetracanthella* spp. **1–2** typical sensillar patterns of western (**1**) and eastern (**2**) groups of species **3–4** labial palp in *T.sylvatica* (**3**) and *T.tardoki* sp. nov. (**4**) Abbreviations: Md, Mdl-dorsal and dorso-lateral macrochaetae.

**S-chaetae on tergites.** In his monograph Deharveng (1987) referred this character to be not of great value to identify the species of the genus. Number of s-chaetae is very conservative in the genus indeed, and probably all species obviously possess 3,3/2,2,2,2,4 s-chaetae, that should be confirmed since this is unknown for several species. The invariable set of s-chaetae is an additional confirmation of the monophyly of the genus. The only key taxonomic character of the genus is four anal spines on Abd.V that is not very safe if considering the independent appearance of spines in the evolution of the family (Deharveng 1978, Potapov et al. 2017). Concerning ms-chaetae, complete set (1,1/1,1,1, Figs 21, 22) is probably shared by almost all species, while, in Far East, *T.orientalis* and *T.tardoki* sp. nov. lost three ms-chaetae resulting 1,0/1,0,0 formula (Figs 46, 47) that is unique for the genus so far. Unlike number, the position of s-chaetae is more variable: the relative position of medial s-chaetae and macrochaetae can differentiate groups of species (Potapov 2001). Medial s-chaetae are situated either behind Mdl macrochaetae (‘*alpina*’, ‘*ethelae*’, ‘*cassagnaui*’, and ‘*wahlgreni*’ groups: Fig. 1) or between Mdl and Ml macrochaetae (‘*grinbergsi*’, ‘*stebaevae*’, and ‘*sylvatica*’ groups: Fig. 2). The former type is a characteristic of European and American groups while the latter one relates to Asiatic ones. This character was not indicated in the descriptions of all species of the genus and exceptions are possible. The European ‘*pilosa*’ group shows rather the “European-American” s-pattern although a few its members show considerable shift of medial s to lateral position. According to figures, at least, in *T.doftana* Fiera, Konikiewicz, Skarżyński, 2013, *T.strenzkei* Gisin, 1949, and *T.gallica* Deharveng, 1987, these s-chaetae are situated behind and lateral to Mdl on abdominal tergites (Deharveng 1987, Fiera et al. 2013).

**Labial palp.** The character is poorly studied for the genus but appears to be promising at least at level of species group. After Fjellberg (1999, Fig. 51) and Smolis and Skarżyński (2006, Figs 12, 32) six *Tetracanthella* species (‘*wahlgreni*’, ‘*alpina*’, and ‘*pilosa*’ groups) from Scandinavia and Poland show reduced A(1)B(3)C(0)D(3)E(5) set in which four guards are lost. In East Asia the species of ‘*stebaevae*’ and ‘*sylvatica*’ groups have complete set [A(1)B(4)C(0)D(4)E(7)], and two young “afurcated” species of ‘*ethelae*’ group lost five guards giving A(1)B(3)C(0)D(3)E(4) formula (Figs 3 and 4, respectively).

### List of the species of *Tetracanthella* of the Far East of Russia (R) and Japan (J)

‘*sylvatica*’ group

*Tetracanthellaannulata* sp. nov. (R)

*Tetracanthellamanschurica* Kutyreva, 1980 (R)

*Tetracanthellasylvatica* Yosii, 1939 (J)

*Tetracanthella* sp. 1 (R)

‘*stebaevae*’ group

*Tetracanthellaczernovae* Kutyreva, 1980 (R)

*Tetracanthella* sp. 2 (J)

‘*ethelae*’ group

*Tetracanthellaorientalis* Martynova in Martynova et al. 1977 (R)

*Tetracanthellatardoki* sp. nov. (R)

‘*wahlgreni*’ group

*Tetracanthellamartynovae* Potapov, 1997 (R)

*Tetracanthellasibirica* Deharveng, 1987 (R)

## Species of the ‘*sylvatica*’ group

### Tetracanthella annulata sp. nov.

Taxon classificationAnimaliaCollembolaIsotomidae

http://zoobank.org/5ABA38DC-0298-4CB7-BC5F-00CDA6CDE57B

Figs 6 , 8–13 , 14–20 , 51 , 58

#### Type material.

Holotype: subadult female, Russia, Far East, Primorye, Terneyski District, Sikhote-Alinski Reserve, Kabani station, 900 m alt., 45.14122°N, 135.87759°E, coniferous forest with *Rhododendronfauriei*, rotten wood, 8.08.2017, leg. N.K., A.G., A.K. Three paratypes: nearly the same place, 932 m alt., 45.13840°N, 135.88702°E, leg. N.K., A.G., A.K.; seven paratypes: Sikhote-Alinski Reserve, Blagodatny station, 95 m alt., 44.96670°N, 136.53410°E, oak forest, rotten wood, 7.08.2017, leg. N.K., A.G., A.K.

#### Other material

(all from the Far East of Russia): **Primorski Krai: Shkotovski district**, Livadiysky Range, Pidan Mt., rotten wood, ~800 m alt., 20.09.2004, leg. M.Potapov; ibidem, trail to Falaza Mt., ~600 m alt., mosses on rotten wood, 08.09.2018, leg. M.P., A.K.; **Primorski Krai, Khasanski district**, “Kedrovaya Pad “ Reserve, valley of Kedrovaya River, cedar litter of mixed forest, 29.09.2004, leg. M.P.; ibidem, 5 km of trail to Central shelter, valley mixed forest, rotten wood, 29.07.2016, leg. N.K., M.P.; ibidem, right bank of Kedrovaya River, 2^nd^ Zolotisti Spring, coniferous litter, 14.07.2013, leg. S. Spiridonov; **Primorski Krai, Lazovsky district**, in mountains nearby Preobrazheniye, Sredni stream (tributary of Maralovaya (valley of Sokolovka River), mixed forest, rotten wood, 21.09.2011, leg. M.P.; **Primorski Krai, Terneyski district**, Ostraya Mt., litter, 02.06–04.06.2018, leg. A.K.; Sikhote-Alinski Reserve, Kabani station, 900 m alt., 45.14122°N, 135.87759°E, coniferous wood with *Rhododendronfauriei*, rotten wood, 8.08.2017, leg. N.K., A.G., A.K.; ibidem, 932 m alt., 45.13840°N, 135.88702°E; Sikhote-Alinski Reserve, Blagodatny station, oak wood, rotten wood, 7.08.2017. 95m alt., 44.96670°N, 136.53410°E; leg. N.K., A.G., A.K. **Primorski Krai, Partyzanski district**, Olkhovaya Mt., 540 m alt., 43.3058°N, 133.6679°E, rotten wood in mixed forest, 20.08.2018, leg. M.P., A.K.

**Khabarovski Krai, Nanaiski District**, Anyuiski National Park, Tormasu River, mixed forest, rotten wood, 204 m alt., 49.30332°N, 137.57004°E, 07.08.2018, leg. N.K., A.G., A.K.; ibidem, Anyuiski National Park, Anyui River, mixed forest, rotten wood, 205 m alt., 49.36350°N, 137.70227°E; Komsomolsk-Khabarovsk road, 270 km, cedarn-large-leaved valley forest, litter, 42 m alt., 048.93659°N, 136.33167°E, leg. N.K., A.G., A.K.; **Khabarovski Krai, Komsomolski District**, Komsomolski Reserve, foothills of Sergol Mt., aspen-oak forest, rotten wood, 259 m alt., 50.73823°N, 137.40182°E, 11.08.2018, leg. N.K., A.G., A.K.; ibidem, Komsomolski Reserve, Sergol Mt., mixed forest with cedar, rotten wood, 228 m alt., 50.73710°N, 137.39772°E, 11.08.2018, leg. N.K., A.G., A.K.; Komsomolski District, Komsomolsk–Khabarovsk road, 85 km, 1,5 km from Gorely Klyuch Stream, mixed forest, rotten wood, 50.21810°N, 137.33202°E, 12.08.2018, leg. N.K., A.G., A.K.

**Amurskaya Region, Arkharinski district**, Khinganski Reserve, 10 km E Uril, coniferous forest, litter, 07.10.2009, leg M.Babykina.

#### Diagnosis.

Coloration spotty, from dark to light grey. Coxa I without an external chaeta. Macrochaetotaxy: 2,2/2,2,2. Dens long, with clear crenulations, without anterior and normally with seven posterior chaetae.

#### Description.

Body length 0.9–1.5 mm. Body cylindrical, not narrowing (Fig. 6). Coloration spotty, from dark to light grey, ventral side of corpus paler, often not pigmented. Pigmentation of antennae vary, paler than other parts of the body, sometimes colorless. Largest polygons much larger than mesochaeta sockets, canals between polygons broad, clearly marked (Fig. 51). No smooth fields. Dorsal mesochaetae long, not shortened in axial part of tergites, in posterior row of Abd. IV not longer than on other parts of body (Md : p1 = 1.8–2.5). Abd. IV with p3 longer than p1 (p3 : p1 = 1.2–1.8) (Fig. 10). Macrochaetae usually blunt and plain at tip.

**Figures 5–7.**
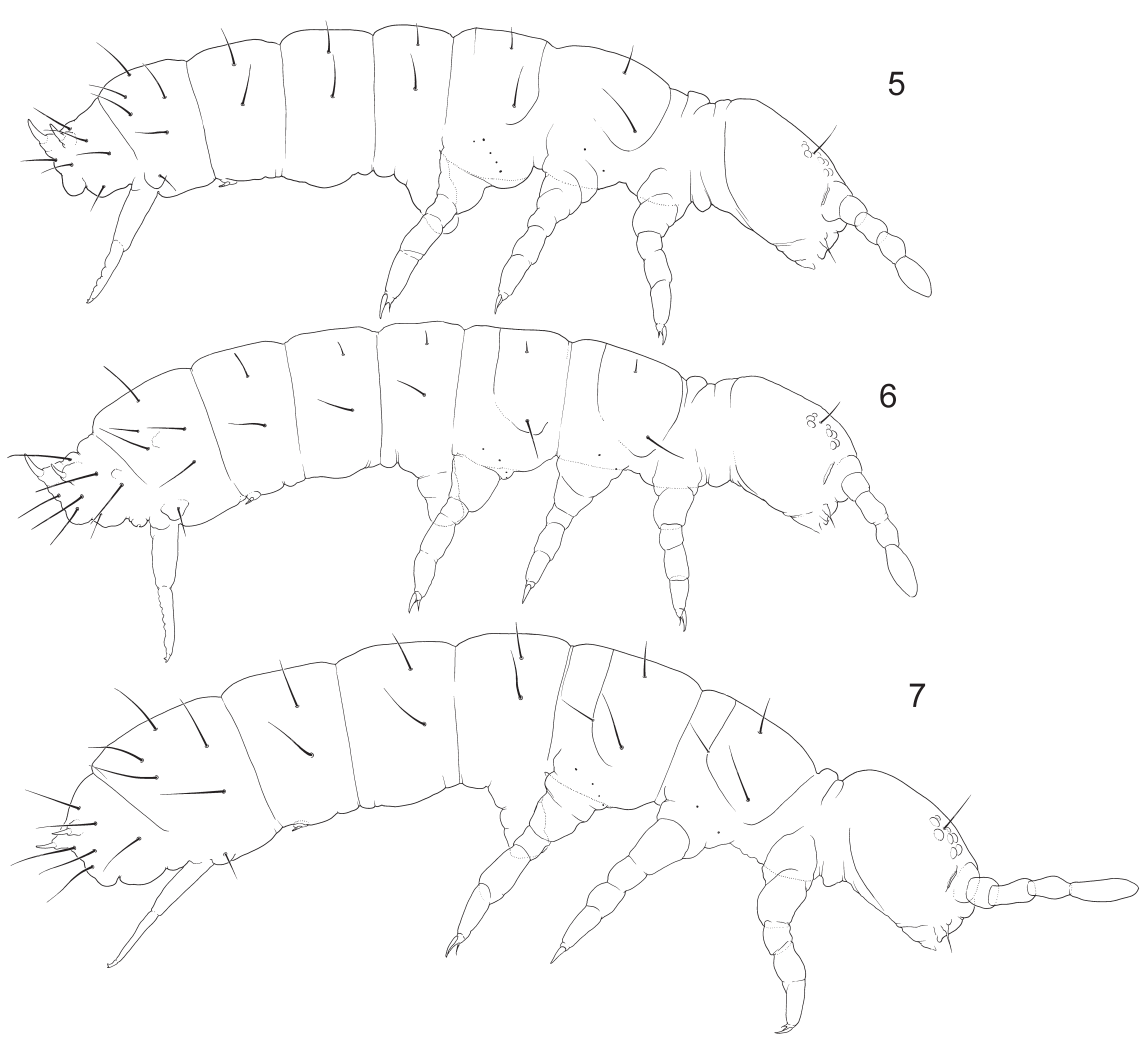
Appearance and macrochaetotaxy of *Tetracanthella* ‘*sylvatica*’ group **5***T.sylvatica***6***T.annulata* sp. nov. **7***T.manschurica*.

8+8 ocelli, G and H reduced (dA : dH = 1.5–2.0). PAO 1.9–2.7 times as long as the diameter of ocellus A (Fig. 16). Chaeta s’ of Ant.III in males absent. Four prelabral chaetae. Outer maxillary lobe with four sublobal hairs and simple maxillary palp. Labium with with complete set of guards [A(1)B(4)C(0)D(4)E(7)], three proximal and four basomedian chaetae. Postlabial chaetae 4+4 (Fig. 11). Five chaetae between medial line and pc3 on head. Frontal chaeta ap present.

**Figures 8–13.**
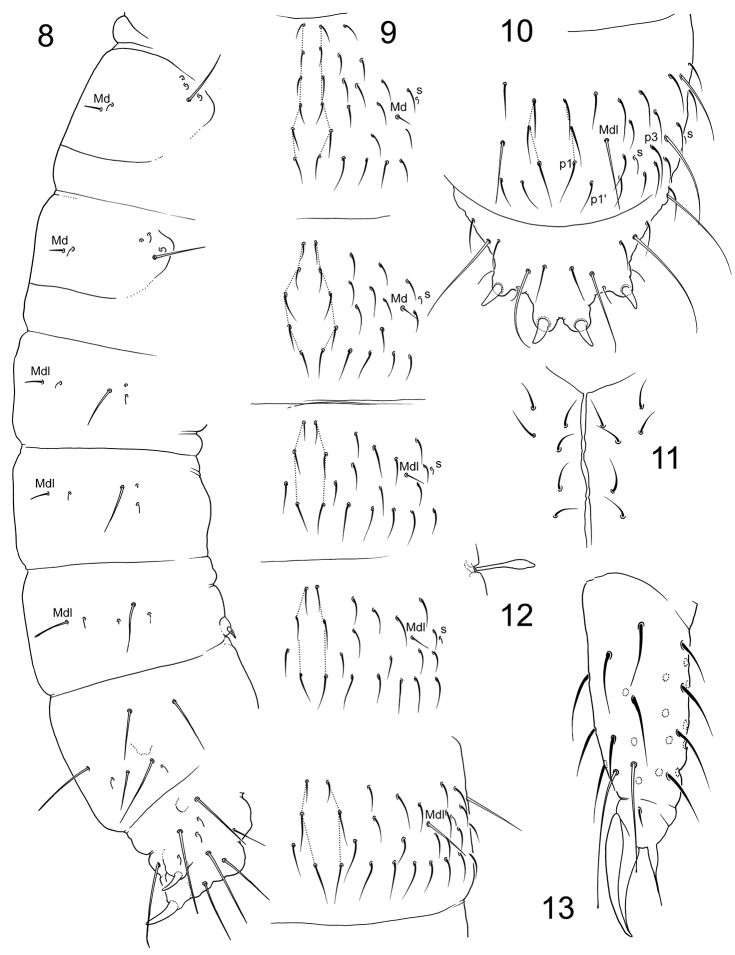
*Tetracanthellaannulata* sp. nov. **8** position of macrochaetae and s-chaetae on corpus **9–10** dorsal chaetotaxy of Th.II–Abd.III (**9**) and Abd.IV (**10**), dorsal view **11** postlabial area **12** spur of Leg 3 in adult male **13** distal part of leg 3.

**Figures 14–20.**
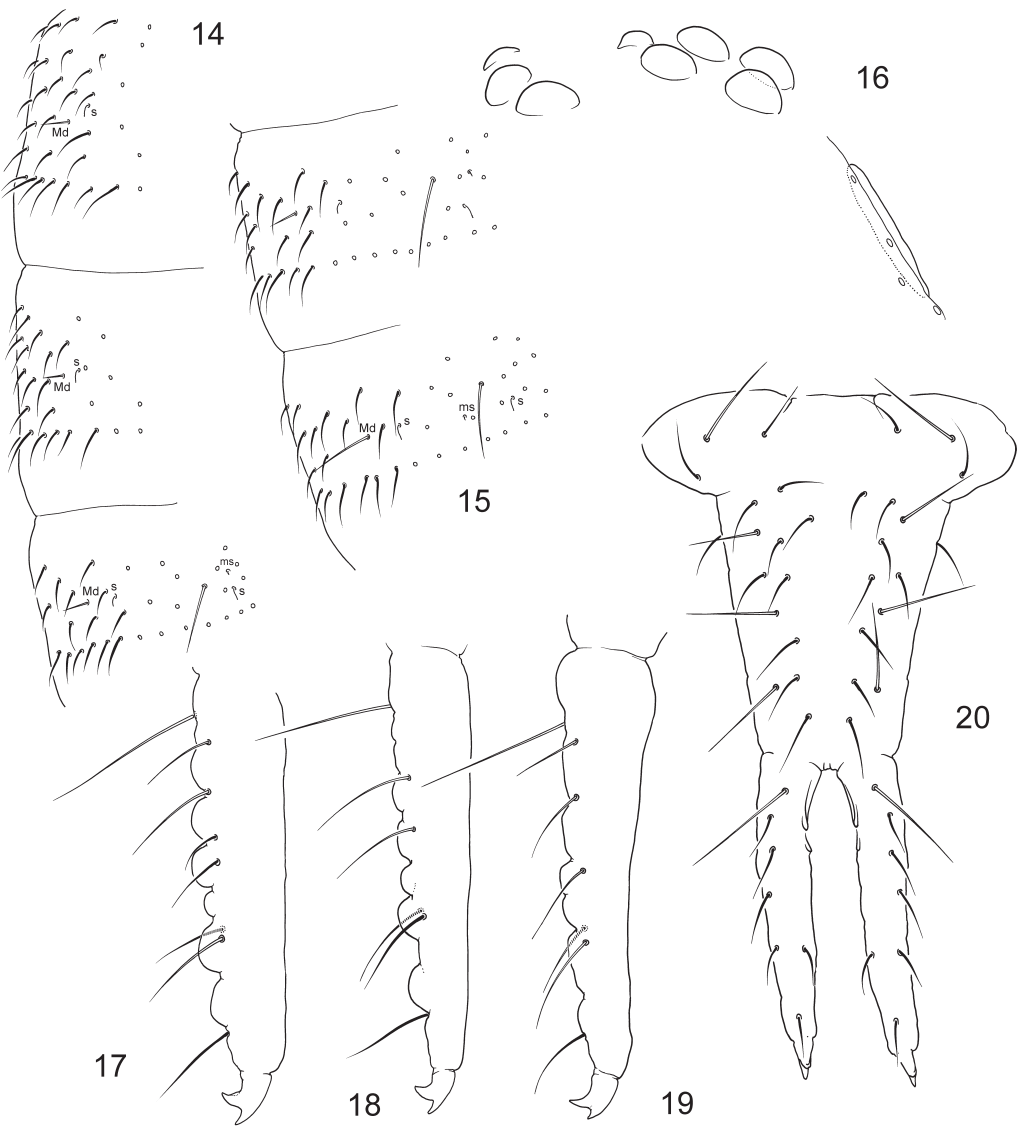
*Tetracanthellaannulata* sp. nov. **14–15** dorsal chaetotaxy of Th.II–Abd.I (**14**) and Abd.II–III (**15**) lateral view **16**PAO and ommatidia **17–19** variation of dens, lateral view **20** furca, posterior view.

Axial chaetotaxy: 12–14,10/6,6,6,6 (without chaetae in Md-position on Abd.I–III and p1’ chaetae on Abd.IV) (Figs 9, 10). Macrochaetotaxy: 2,2/2,2,2. Md macrochaetae on Th.II and III and Mdl macrochaetae on Abd.I and II short (Figs 8, 14, 15). Corner mesochaetae on Th. II and III not stronger than other mesochaetae of p-row. Number of s-chaetae: 3,3/2,2,2,2,4 (s), 1,1/1,1,1 (ms) (Fig. 8). S-chaetae of medium size, medial ones on Abd. I–III arranged lateral to Mdl macrochaetae. Sternite of Th. III without chaeta.

Coxa I without an external chaeta. Tibiotarsi with 1,2,2 clavate dorsal tenent hairs (Fig. 13) and 1,1,0 ventral pointed long hairs. Males with chaeta B5 and X on tibiotarsi III expanded, spatula-like (Fig. 12), these chaetae thin in females. Tibiotarsi I, II, III with 21, 21, 25 chaetae. Claw untoothed, empodial appendage long, 0.6–0.8 as long as inner edge of claw, with long apical filament (Fig. 13). Ventral tube with 3+3 latero-distal and four posterior chaetae.

Retinaculum with a chaeta and 4+4 teeth, basal tooth smaller. Anterior furcal subcoxa with 8–9 (rarely seven or ten) chaetae, posterior one with 5–6 (rarely four or seven) chaetae. Dorsal side of manubrium with 3+3 laterobasal chaetae and 11+11 (sometimes ten or 12 on one side) chaetae on main part (14+14 at whole), besides with a chaeta on each lateral side (Fig. 20). Mucro bidentate, small. Dens long, always with clear crenulations, without anterior and with 6–8 (normally 7, rarely 9) posterior chaetae (Figs 17–19). Dens : claw III = 3.5–4.3. Manubrium : dens : mucro = 8–12 : 8–12 : 1.

Anal spines parallel, large, on moderate papillae. Medial mesochaetae (a1) of Abd. V slightly in front of medial macrochaetae (a2). Arrangement of chaetae and spines on dorsum of Abd V as a2-a2/a1-a1 =2.1–2.3; a2-a2/a2-eAS = 1.7–1.8 (Fig. 10). Males present.

#### Etymology.

The species is characterized by annulated posterior side of dens.

#### Distribution and ecology.

The species is widely distributed in southern areas of the Far East of Russia (Primorsky Krai, Khabarovsky Krai and Amurskaya District), both in flatland and in the mountains (Fig. 58). It prefers rotten wood although occurs in forest litter.

#### Discussion.

The new species belongs to ‘*sylvatica*’ group and differs from all species of the group by absence of chaetae on anterior side of dens. The disproportion of anterior and posterior number of chaetae on dens (0 vs. ~7), clear humps on posterior side of dens and grey coloration make *T.annulata* sp. nov. unmistakable in the area of its distribution.

### Tetracanthella sylvatica

Taxon classificationAnimaliaCollembolaIsotomidae

Yosii, 1939

**Figures 21–22.**
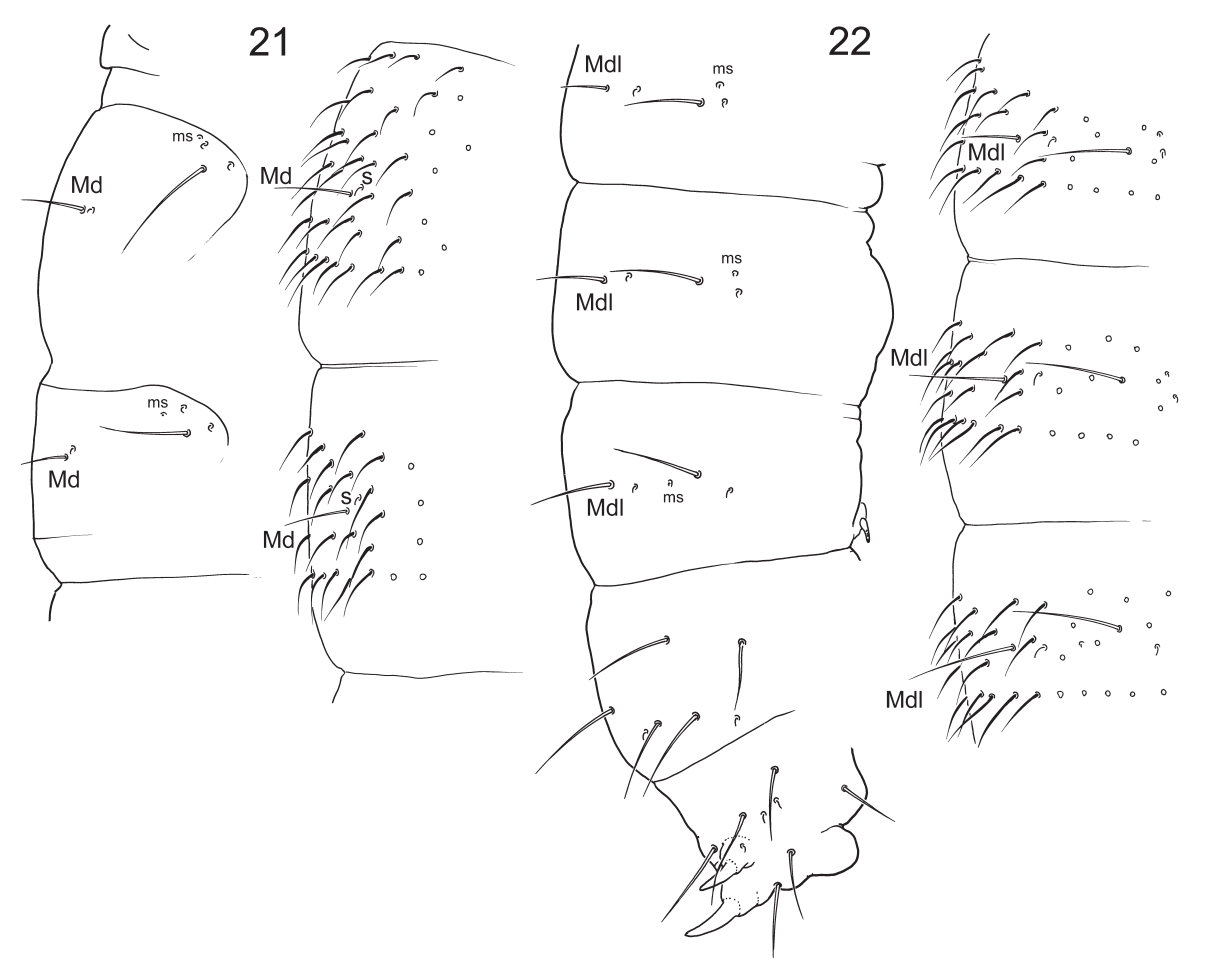
*Tetracanthellasylvatica*, position of macrochaetae and s-chaetae on thorax (**21**) and abdomen (**22**).

Figs 3 , 5 , 21 , 22 , 52 , 58

#### Material.

Japan, Honshu, Kyoto, Kamigamo experimental forest in Kyoto University, 2011, leg. S. Fujii.

#### Distribution.

*Tetracanthellasylvatica* was described from Osaka (central Honshu) and was numerously recorded around here afterwards, particularly from Kamigamo Experimental Forest of Kyoto University (e.g., Takeda 1973, Deharveng 1987, Fujii et al. 2014). A few records are known from Shikoku and more northern areas of Honshu (Yosii 1969, Tamura and Chiba 1977, Yamauchi and Suma 1999, 2009, Nakamura et al. 2006, Niijima 1976) (Fig. 58). The species was recorded once from Hokkaido (Suma 1990).

#### Discussion.

The remarks to chaetotaxy of the species were given by Yosii (1961), the complete redescription was provided by Deharveng (1987). After our materials, the species has complete set of guards in labial palp (Fig. 3) that is common for the species of eastern groups (‘*sylvatica*’, ‘*stebaevae*’, ‘*grinbergsi*’). *Tetracanthellasylvatica*, *T.annulata* sp. nov., and *T.dorsoduplex* Xie, Potapov, Sun, 2019 combine a natural group of species distributed in East Asia. They share long furca with annulated dorsal side of dens, well developed reticulation with broad canals between polygons (Figs 51–52), and few macrochaetae on body tergites. *Tetracanthellasylvatica* differs from other two species by better development of medial macrochaetae on body (Figs 5, 21, 22), from *T.annulata* sp. nov. by the presence of anterior chaeta on dens, from *T.dorsoduplex* by common position of lateral s on Abd.IV. The specimens from Kamigamo have 3+3 postlabial chaetae that could be an additional differentiated character of the species if confirmed by wider Japanese materials.

In the alpine zone of Ezop Range (western part of Khabarovski Krai, leg. A.B.) we discovered a form which shares many essential characters with typical *T.sylvatica* from which it differs by larger body (~2 mm), 4+4 postlabial chaetae and absence of annulations on posterior side of dens.

### Tetracanthella manschurica

Taxon classificationAnimaliaCollembolaIsotomidae

Kutyreva, 1980

Figs 7 , 23–25 , 26–35 , 53 , 58

#### Material

(all from the Far East of Russia): Khabarovski Krai, Imeni Lazo district, upper flux of Katen River, Ko Mt., upper part of Ko Stream, ~970 м alt., 29.06.2018, soil in coniferous forest, A.B.; Khabarovski Krai, Vaninski district, ~14 km N Vysokogorny, upper flux of Mulinka River, closed spruce forest at pass, ~900 m alt., 29.09.2011, leg. M.P.; Primorski Krai, Partyzanski district, Olkhovaya Mt., 1380 m alt., 43.3375°N, 133.6615°E, spruce litter, 20.08.2018, leg. M.P., A.K.; Primorski Krai, Chuguevski district, Oblachnaya Mt., 1230 m alt., 43.6483°N, 134.1978°E, spruce litter, 19–20.09.2018, leg. A.K.; Primorski Krai, Chuguevski district (unprecise locality), spruce forest, 8.09.1973, leg. L. Kutyreva.

#### Description based on the aforementioned specimens.

Body length 1.6–1.9 mm. Body thick, tubular, not narrowed (Fig. 7). Coloration dark blue, distal half of antennae white. Reticulation very thin, polygons much smaller than mesochaeta socket (Fig. 53). No smooth fields. Size of dorsal mesochaetae variable (see the Remarks part), not shortened in axial part of tergites (Md : p1 = 1.3–1,5). Abd. IV with p3 subequal to p1. Macrochaetae acuminate.

8+8 ocelli, G and H smaller. PAO short, 1.1–1.6 as long as the diameter of ocellus A (Fig. 28). Chaeta s’ of Ant.III in males absent. Four prelabral chaetae. Outer maxillary lobe with four sublobal hairs and simple maxillary palp. Labium with complete set of guards [A(1)B(4)C(0)D(4)E(7)], three proximal and four basomedian chaetae. Postlabial chaetae 4–5+4–5. With 8–10 chaetae between medial line and pc3 on head. Frontal chaeta ap present.

**Figures 23–25.**
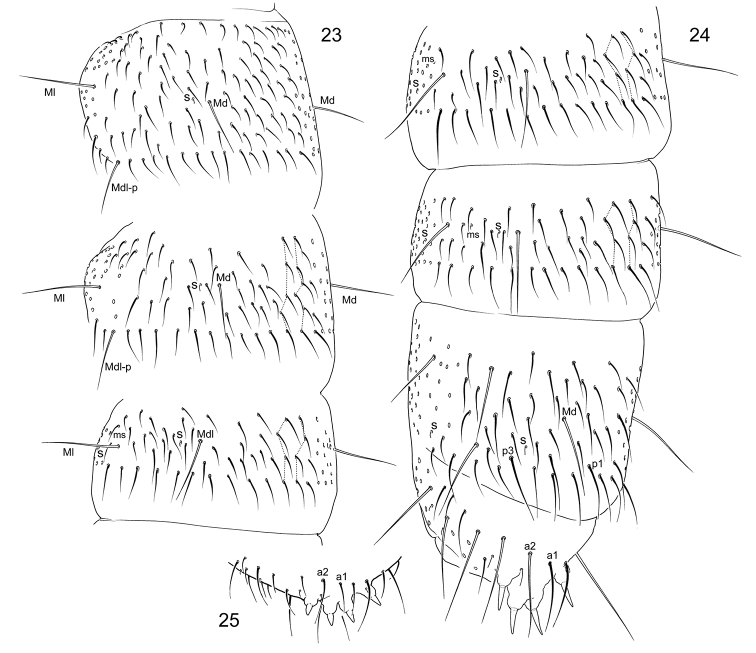
*Tetracanthellamanschurica***23–24** dorsal chaetotaxy, dorsal view (Vaninski district) **25** anal spines (Chuguevski district).

Chaetotaxy abundant (Figs 23, 24). Axial chaetotaxy often asymmetrical 12–14,8/8,8,8,8–10 Macrochaetotaxy: 3(W),3(W)/2,2,2 (Fig. 27). Mdl macrochaetae in p-row on Th. II and III. Number of s-chaetae: 3,3/2,2,2,2,4 (s), 1,1/1,1,1 (ms) (Fig. 27). S-chaetae short, medial ones on Abd. I–III arranged lateral to Mdl macrochaetae. Sternite of Th. III without chaetae.

Coxa I without an external chaeta. Tibiotarsi with 1,2,2 long and clavate dorsal tenent hairs and without well developed ventral tenent hairs. Tibiotarsi with many additional chaetae on all legs, tibiotarsi I and II with 26–28 chaetae each, III with more than 30 chaetae (Fig. 34). Empodial appendage 0.7–0.8 as long as inner edge of claw, with apical filament.

**Figures 26–35.**
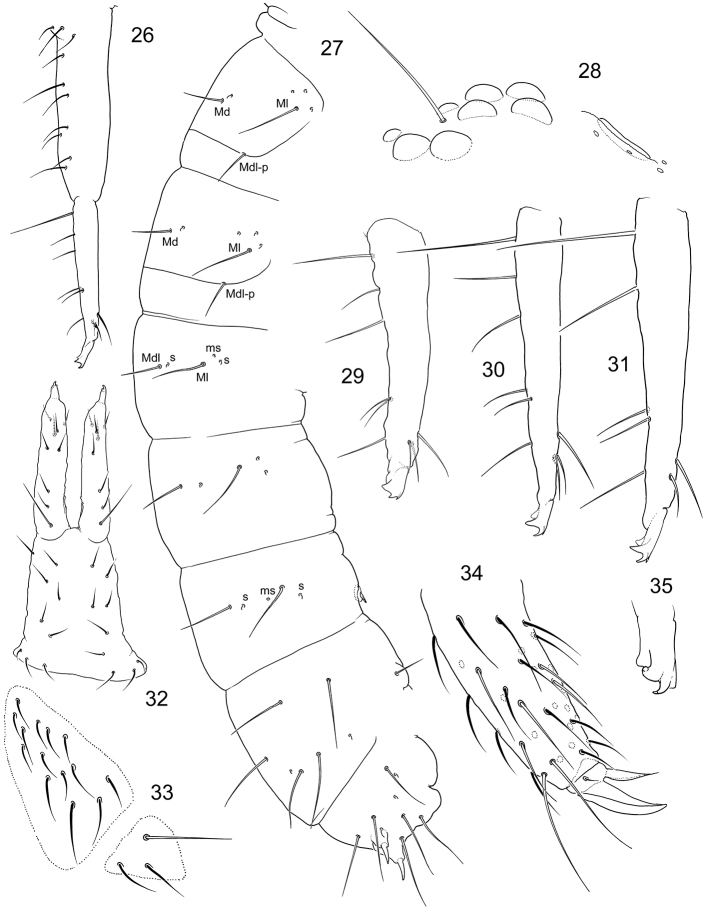
*Tetracanthellamanschurica***26** furca, lateral view (Vaninski district) **27** position of macrochaetae and s-chaetae on corpus **28**PAO and ommatidia **29–31** dens, lateral view in specimen from Imeni Lazo district (**29**) and Vaninski district (**30, 31**) **32** furca, posterior view, juvenile specimen **33** furcal subcoxae **34** distal part of leg 3 **35** mucro.

Ventral tube with 3+3 laterodistal and four posterior chaetae. Retinaculum with 4+4 teeth and a chaeta. Anterior furcal subcoxa with 10–17 chaetae, posterior one with 3–4 chaetae (Fig. 33). Posterior side of manubrium with 6(7)+6(7) chaetae on main part and 3+3 on basolateral parts (9+9 at whole) (Figs 26, 32). Mucro indistinctly bidentate, with two teeth and some lamellae which make illusion of tridentate or quadridentate mucro (Figs 26, 29–32, 35). Anterior side of dens with three anterior chaetae, one larger and in more distal position and two (rarely one) smaller on both its sides. Posterior side with six (rarely five) chaetae, arranged as 1+1+(1)+2+1 (Figs 29–31). Dens:claw III = 3.1–4.8 (see the Remarks part). Manubrium : dens : mucro = 6.8–9.3 : 4.6–7.1 : 1. Inner and outer anal spines parallel, relatively small, on unsclerotised high papillae. Medial mesochaetae (a1) of Abd. V at level or slightly posterior to medial macrochaetae (a2) (Figs 24, 25). Arrangement of chaetae and spines on dorsum of Abd V as a2-a2/a1-a1 ~3.0; a2-a2/a2-eAS ~1.7. Males present.

#### Distribution and ecology.

*Tetracanthellamanschurica* occurs in the mountains of Sikhote-Alin Range (Fig. 58). It is a rare species preferring coniferous litter.

#### Discussion.

*Tetracanthellamanschurica* was described from Lazovski district of Primorski Krai. Afterwards, it was recorded once with few morphological remarks by Potapov (2001). The only known type individual of the species is probably lost and our redescription is based on 15 specimens from three different districts of the same region. These specimens share several peculiar features and generally fit to the original description. Some discrepancy between the text of the first description and our observations is probably due to the juvenile condition of the holotype (Kutyreva 1980). *Tetracanthellamanschurica* belongs to the ‘*sylvatica*’ group and differs from other species of the group (*T.sylvatica*, *T.annulata* sp. nov., and *T.dorsoduplex* by three chaetae on anterior side of dens, absence of crenulation on posterior side of dens, thin reticulation of cuticle, dark blue colouration and few chaetae on posterior side of manubrium and posterior furcal subcoxa. *Tettracanthellamanschurica* additionally has very peculiar mucro which appears to have three or four teeth due to one or two lamellae. Regardless the lamellae, mucro of this species keeps the general bidentate pattern known in the genus.

Population from the northern part of Sikhote-Alin Range (Vaninski district) show longer meso- and macrochaetae (Fig. 24), clearly clavate tenent hairs on legs, longer claws (dens:claw III = 3.1–3.6) and mucro (dens : mucro = 4.6–5.1 : 1) (Figs 30, 31). More southern populations (Fig. 58) (correspond better to the type specimens morphology because of shorter meso- and macrochaetae (Fig. 25), slightly (vs. clearly) clavate tenent hairs, short claws (dens : claw III = 4.1–4.8) and short mucro (dens : mucro = 6.7–7.1 : 1) (Fig. 29). We include both forms to the diagnosis of *T.manschurica* in view of the possible ecomorphic nature of the differences. One individual of unclear status from Kunashir Island (Alekhino, leg. I. Volonikhina) differs from continental populations by much shorter dens.

## Species of the ‘*stebaevae*’ group

### Tetracanthella czernovae

Taxon classificationAnimaliaCollembolaIsotomidae

Kutyreva, 1980

Figs 36–39 , 54

#### Type material.

Lectotype and one paralectotype (females) designated and labeled as: Primorski Krai: Shkotovski district, NE part of Livadiysky Range, Krinichnaya (= Falaza) Mt., coniferous forest belt with *Picea* and *Abies*, litter under *Abiesnephrolepis*, 12.10.1977, leg. L. Kutyreva

#### Redescription.

Body length 2.0 mm (for subadult female). Body thick, tubular. Coloration dark, antennae white. Reticulation thin, polygons smaller than mesochaeta socket (Fig. 54). No smooth fields. Dorsal mesochaetae long, not shortened in axial part of tergites, (Md : p1 = 1.2–1,4). Abd. IV with p3 subequal to p1. Macrochaetae acuminate.

8+8 ocelli, G and H smaller. Four prelabral chaetae. Outer maxillary lobe with four sublobal hairs and simple maxillary palp. Labium with complete set of guards [A(1)B(4)C(0)D(4)E(7)], three proximal and four basomedian chaetae. Postlabial chaetae 4+4. With 7–8 chaetae between medial line and pc3 on head (Fig. 37). Frontal chaeta ap present.

**Figures 36–39.**
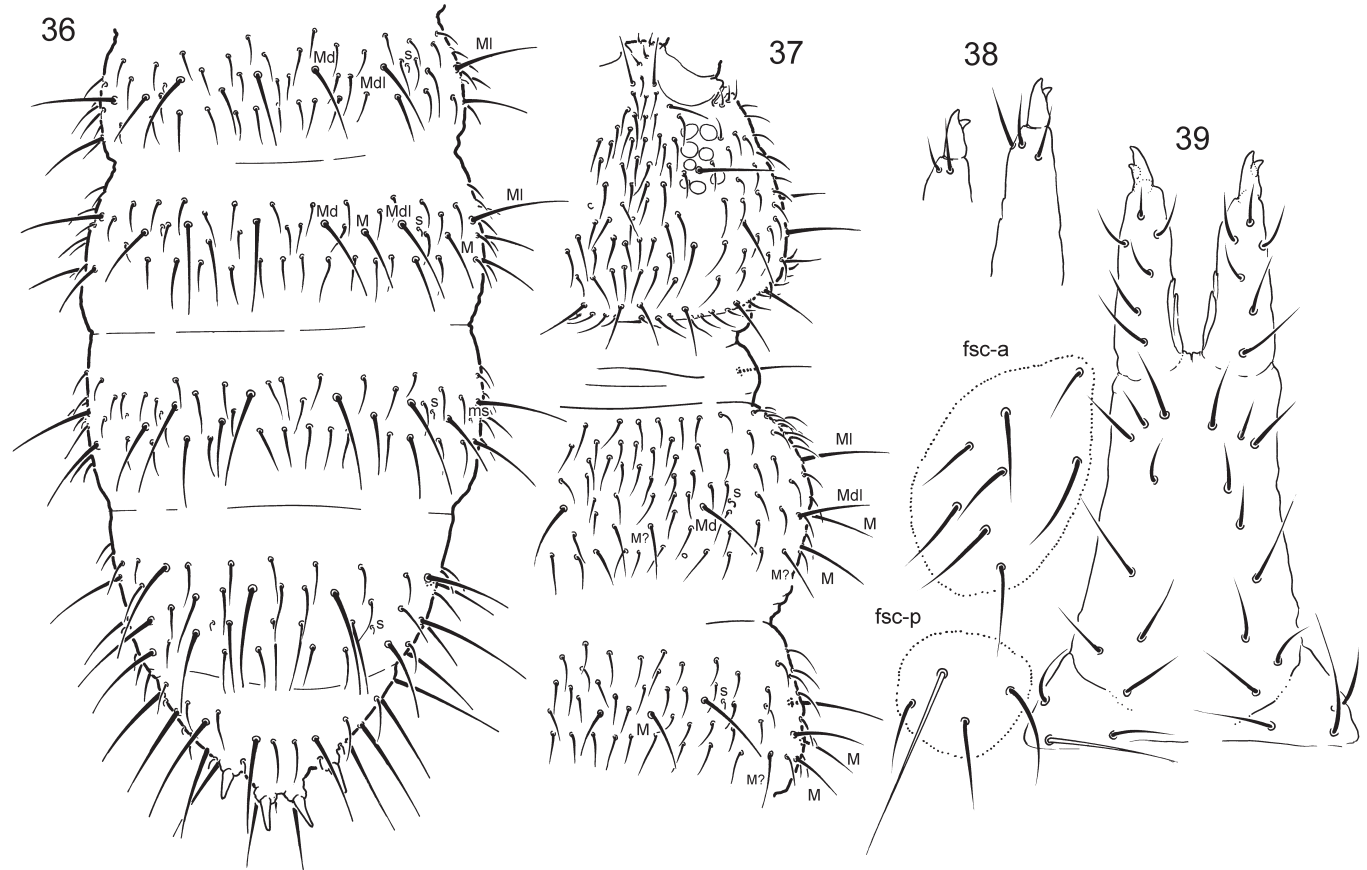
*Tetracanthellaczernovae***36–37** dorsal chaetotaxy of thorax (**36**) and abdomen (**37**), dorsal view **38** anterior side of dens, paralectotype (left) and lectotype (right) **39** furcal area, lectotype. Abbreviations: fsc-a and fsc-p, anterior and posterior furcal subcoxae.

Chaetotaxy abundant (Figs 36, 37). Axial chaetotaxy 12–14,8/6,6,6,6. Basic set of macrochaetae complete: 3(A),3(A)/3,3,3. Besides, additional macrochaetae present posterior to basic macrochaetae resulting full formula 3+’4’,3+’4’/3+’3’,3+’3’,3+’3’. Some chaetae of p-row also macrochaeta-like. Number of s-chaetae: 3,3/2,2,2,2,4 (s), 1,1/1,1,1 (ms). S-chaetae short, medial ones on Abd. I–III arranged lateral to Mdl macrochaetae. Sternite of Th. III without chaetae.

Coxa I without an external chaeta. Tibiotarsi with 1,2,2 long and clavate dorsal tenent hairs. Ventral tenent hairs weakly developed. Tibiotarsi I, II, III with 21, 21, 25 chaetae, respectively. Empodial appendage 0.7–0.8 as long as inner edge of claw, with apical filament.

Ventral tube with 3+3 laterodistal and four posterior chaetae. Retinaculum with 4+4 teeth and a chaeta. Anterior furcal subcoxa with 8–9 chaetae, posterior one with four chaetae (Fig. 39). Posterior side of manubrium with 8–9+8–9 chaetae on main part and 3+3 on basolateral parts (Fig. 39). Mucro bidentate, short. Anterior side of dens with two or three chaetae (differing in lectotype and paralectotype) (Fig. 38). Posterior side with six chaetae. Dens : claw III = 1.4–1.6. Manubrium : dens : mucro = 9–12 : 4–5 : 1. Inner and outer anal spines parallel, on high unsclerotised papillae. Medial mesochaetae (a1) of Abd. V slightly posterior to medial macrochaetae (a2). Arrangement of chaetae and spines on dorsum of Abd V as a2-a2/a1-a1 ~2.8; a2-a2/a2-eAS ~1.7. Males unknown.

#### Distribution.

The species is known only from type locality, by two specimens.

#### Discussion.

*Tetracanthellaczernovae* belongs to the ‘*stebaevae*’ group due to chaeta on coxa I missing and complete set of macrochaetae on tergites. The species however shares many essential characters, incl. appearance, with *T.manschurica* (‘*sylvatica*’ group). *Tetracanthellaczernovae* was briefly redescribed by Potapov (2001) from two specimens supposed to be syntypes. These two specimens from the collection of E. Kutyreva did not have labels indicating type status. We designate two specimens collected by L. Kutyreva as lectotype and paralectotype. *Tetracanthellaczernovae* resembles *T.wui* Xie, Potapov, Sun, 2019 but differs by having more setae on the dens (2–3/6 vs. 1/5).

One individual from central Honshu (Japan, Nagano Prefecture: Chino, leg. M.P. and N.K.) is close to *T.czernovae* but obviously represents a new species differing by absence of additional macrochaetae on body and presence of additional chaetae on Tibiotarsi I and II. It is the second species of the genus *Tetracanthella* occurring in Japan.

## Species of the ‘*ethelae*’ group

### Tetracanthella tardoki sp. nov.

Taxon classificationAnimaliaCollembolaIsotomidae

http://zoobank.org/034F2534-C6F0-4F1B-B83E-AE022A1496C3

Figs 4 , 41 , 42–45 , 46 , 55–56 , 58

#### Type material.

Holotype: female, Russia, Far East, Khabarovsky Krai, Nanaisky district, ~40 km S road Khabarovsk-Sov.Gavan, Tardoki-Yani Mt., ~2050 m alt., tundra on top, 16–26.06.2017, leg. A.B. 19 paratypes from the same place and nearby, 1800–1900 m alt.

#### Other material

(all from Tardoki-Yani Mt.): different open sites nearby type locality (moss and lichen on talus, mountain tundra, and mosses on rocks), 16–26.06.2017, leg. A.B.

#### Diagnosis.

Coxa I without an external chaeta. Macrochaetotaxy: 3(W),3(W)/2,3,3. Retinaculum and furca absent.

#### Description.

Body length 1.2–1.7 mm. Body slender, continuously narrowing (Fig. 41). Coloration dark, including antennae. Polygons large, canals between polygons well-marked. Smooth fields present on Abd.II–IV (Fig. 56), often on Abd.I. Head (Fig. 55), Th.II and III sometimes with narrow smooth belts at posterior edge. Area between ASi sometimes with small field. Dorsal mesochaetae rather short, slightly shortened in axial part of tergites (Fig. 43), in posterior row of Abd. IV not longer than on other parts of body (Md : p1 = 5.7–7.8). Abd. IV with p3 much longer than p1 (p3 : p1 = 3.4–4.5). Macrochaetae long and thick.

**Figures 40–41.**
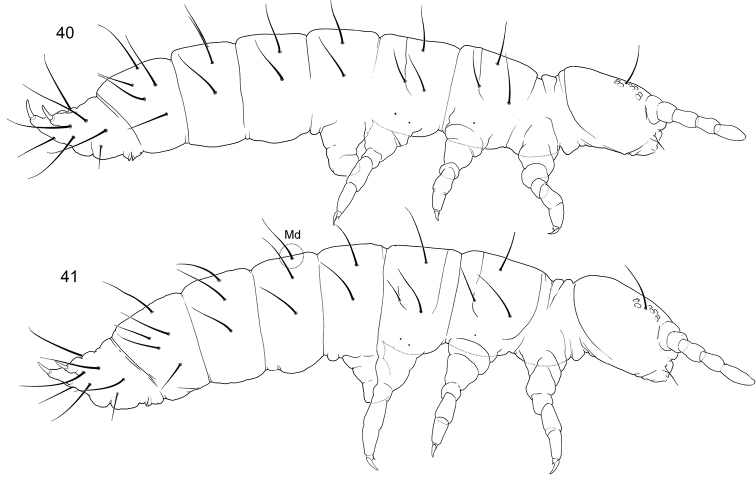
Appearance and macrochaetotaxy of *T.orientalis* (**40**) and *T.tardoki* sp. nov. (**41**).

8+8 ocelli, G and H reduced. PAO 2.5–3.3 as long as the diameter of ocellus A. Chaeta s’ of ant.III in males present. Two prelabral chaetae. Outer maxillary lobe with three sublobal hairs and simple maxillary palp. Labium with three proximal and four basomedian chaetae, labial palp with reduced set of guards [A(1)B(3)C(0)D(3)E(4)] (Fig. 4): papillae B and D each lost one dorsal guard (b4 and d4, respectively), papilla E lost three guards (e7 and probably e5 and e3). Postlabial chaetae 3+3. With 4–5 (rarely three in smaller and juvenile individuals) chaetae between medial line and pc3 on head. Frontal chaeta ap absent.

**Figures 42–45.**
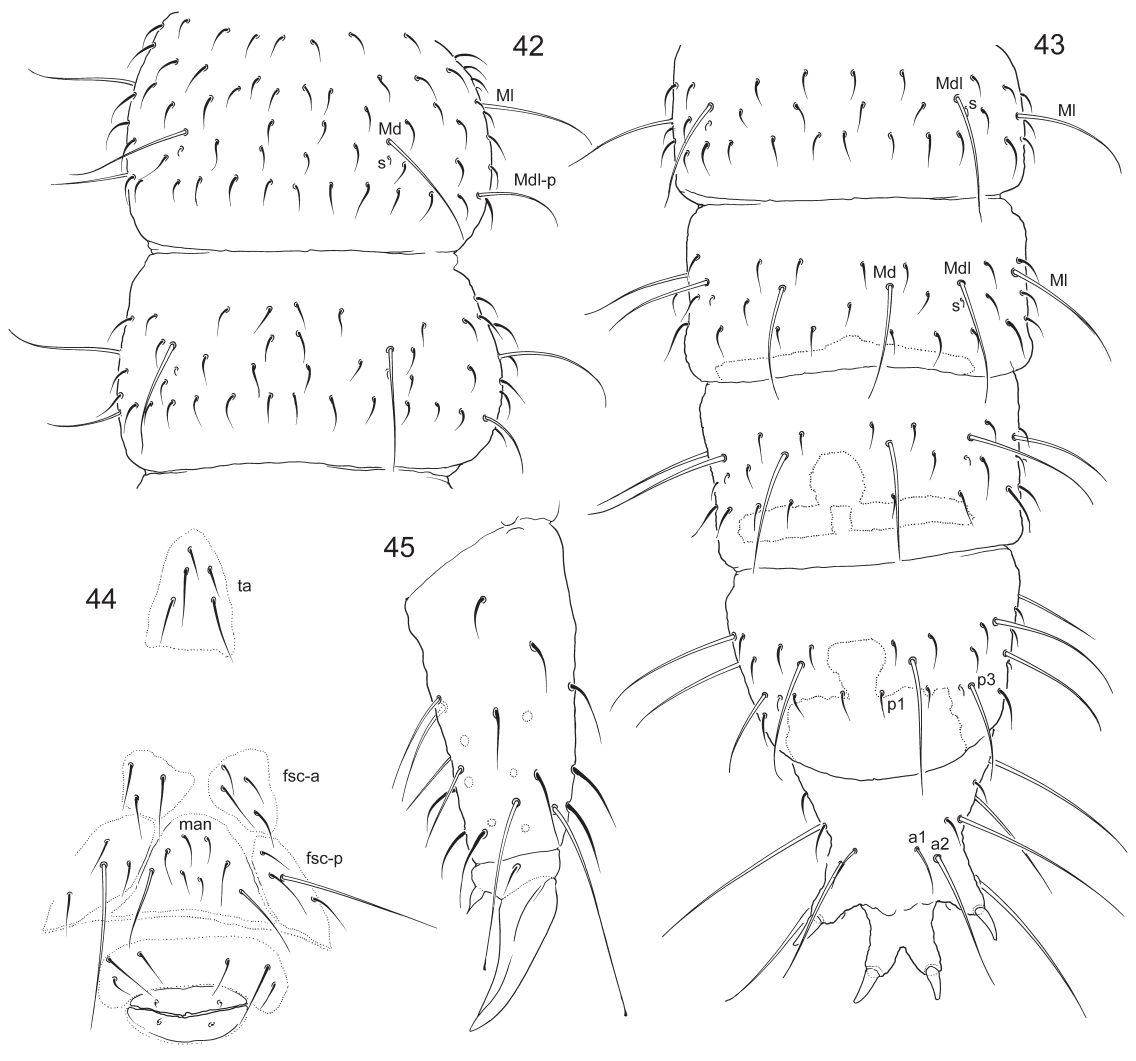
*Tetracanthellatardoki* sp. nov. **42–43** dorsal chaetotaxy of thorax (**42**) and abdomen (**43**), dorsal view **44** tenacular, furcal and genital areas of female **45** distal part of leg 3. Abbreviations: fsc-a and fsc-p–anterior and posterior furcal subcoxae, man manubrial field, ta tenacular area.

**Figures 46–50.**
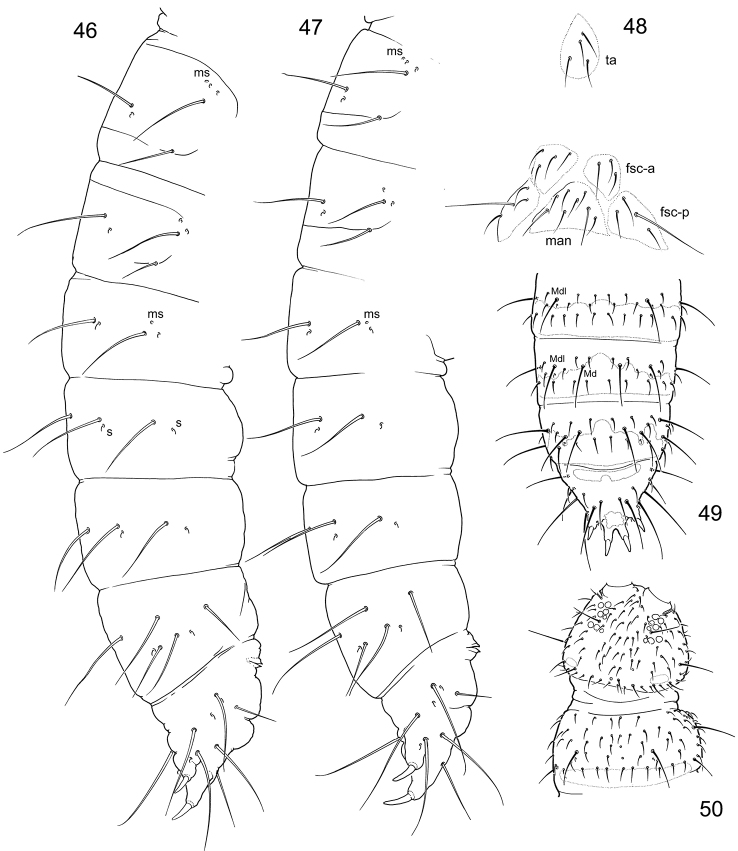
*Tetracanthellatardoki* sp. nov. (**46**) and *T.orientalis* (**47–50**) **46–47** position of macrochaetae and s-chaetae on corpus **48** tenacular and furcal areas **49–50** dorsal chaetotaxy of Abd.II–V (**49**), head and Th.II (**50**), dorsal view. Abbreviations: fsc-a and fsc-p anterior and posterior furcal subcoxae, man manubrial field, ta tentacular area.

Chaetotaxy scarce (Figs 42, 43). Axial chaetotaxy 10,8/4,4,4,4. Macrochaetotaxy: 3(W),3(W)/2,3,3. Mdl macrochaetae in p-row on Th. II and III. Number of s-chaetae: 3,3/2,2,2,2,4 (s), 1,0/1,0,0 (ms) (Fig. 46). S-chaetae short, medial ones on Abd. I–III arranged behind Mdl macrochaetae. Sternite of Th. III without chaeta.

Coxa I without an external chaeta. Tibiotarsi with 1,2,2 long and clavate dorsal tenent hairs and 3,3,1 enlarged ventral tenent hair (Fig. 45). Males with chaeta B5 and X on tibiotarsi III stick-like, thickened. Tibiotarsi I and II with 21 chaetae each, III with 22 chaetae. Claw without teeth. Empodial appendage very short, 0.15–0.20 as long as inner edge of claw (Fig. 45).

Ventral tube with 3+3 lateral and four posterior chaetae.

Retinaculum and furca absent. Retinacular field with 3–5 chaetae. Anterior furcal subcoxa with three (rarely two or four) chaetae, posterior one with four chaetae. Manubrial field normally with eight chaetae (Fig. 44). Anal spines parallel, large, on high papillae. Papillae of inner pair sclerotised. Medial mesochaetae (a1) of Abd. V anterior to medial macrochaetae (a2) (Fig. 43). Arrangement of chaetae and spines on dorsum of Abd V as a2-a2/a1-a1 = 1.6–1.8; a2-a2/a2-eAS = 1.5–2.0 (Fig. 43). Males present.

#### Etymology.

The species is named after the type locality.

#### Distribution and ecology.

It is known only from the Tardoki-Yany mountain massive (central part of Sikhote-Alin Range) where it occurs in all samples from alpine sites which we have examined (Fig. 58).

#### Discussion.

The new species belongs to the ‘*ethelae*’ group by absence of chaeta on coxa I, three sublobal hairs, two prelabral chaetae and other characters. Together with *T.orientalis* they are the only representatives of this Nearctic group in Palearctic. The two species share several apomorphic characteristics unknown in North American species: absence of furca, presence of the third macrochaetae in p-position on thorax, low number of axial chaetae, short empodium. *Tetracanthellatardoki* sp. nov. differs from *T.orientalis* by the presence of Md macrochaetae on Abd.II resulting in formula 2,3,3 (vs. 2,2,3) on abdomen.

### Tetracanthella orientalis

Taxon classificationAnimaliaCollembolaIsotomidae

Martynova, 1977 in Martynova et al. 1977

Figs 40 , 47–50 , 57 , 58

#### Material.

**Magadanskaya region**: vicinities of Magadan, Snow Valley, 18.09.1974. It is the type locality of the species although the type specimens were not seen by us and are probably lost.

**Chukotski AO**: Anadyrski district, vicinities of Anadyr, Observatsii Cape, tundra, 27.06.1974, leg. E. Bondarenko, Anadyrski district, Ugolnaya Bay, tundra, leg. M. Chernyakhovski.

**Kamchatka**: Yuzhno-Kamchatski Reserve, Elizovski district (south), Kambalnoye Lake, pine elfin wood, 14.09.2005, leg. L. Lobkova; Elizovski district (north), Kronotski reserve, caldera of Uzon, moss-lichen tundra, 20.08.2005, leg. L. Lobkova; Kronotski reserve, Vachkazhets Volcano, 1000 ma lt., tundra, gopher burrow, leg. L. Lobkova; Bystrinski district, vicinities of Anavgai and Esso, 3–5.07.2012, tundra at lake (*Ledum, Empetrum*), leg. M.P.

#### Description.

Body length 1.2–1.6 mm. Body slender, continuously narrowing (Fig. 40). Coloration dark, including antennae. Polygons large, canals between polygons well marked. Smooth fields usually present on all tergites of body (Fig. 49, 50). Posterior edge of head with smooth fields in lateral position (Fig. 50) or two groups of larger polygons (Fig. 57) in associated places. Area between ASi often with small smooth field. Dorsal mesochaetae short, slightly shortened in axial part of tergites (Fig. 49), in posterior row of Abd. IV not longer than on other parts of body (Md:p1 = 3.7–5.7). Abd. IV with p3 much longer than p1 (p3 : p1 = 2.3–4.4). Macrochaetae long.

**Figures 51–57.**
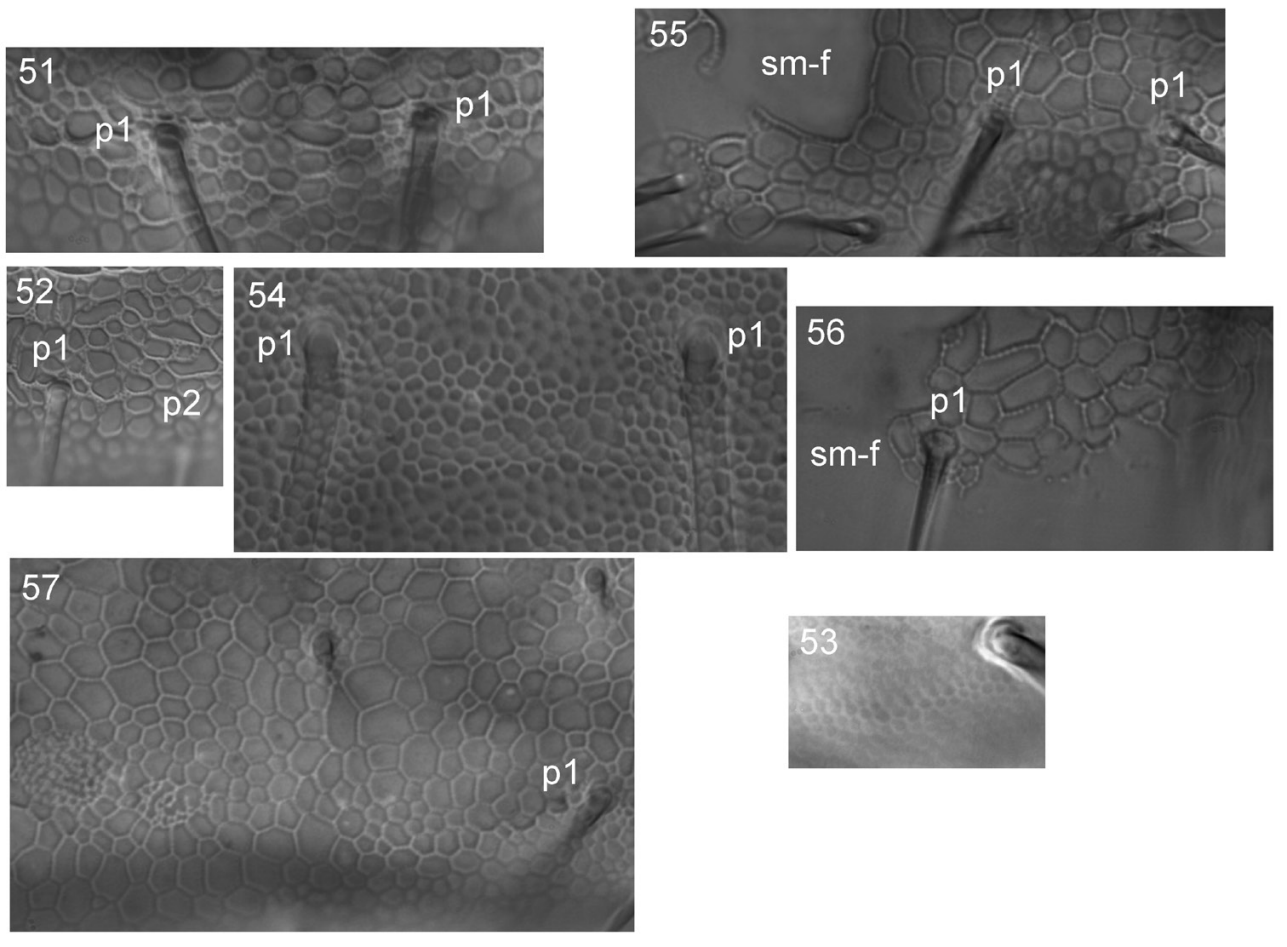
Reticulation of cuticle in *Tetracanthella* of East Asia **51***T.annulata* sp. nov., posterior edge of Abd.IV **52***T.sylvatica*, ibidem **53***T.manschurica*, posterior edge of Abd.IV, lateral part **54***T.czernovae*, posterior edge of Abd.IV **55–56***T.tardoki* sp. nov., posterior edge of head (**55**) and posterior edge of Abd.IV (**56**) **57***T.orientalis*, posterior edge of head. Abbreviations: p1 and p2, chaetae of p-row, sm-f smooth field.

8+8 ocelli, G and H reduced (dA : dH = ~1.5). PAO 2.5–4.0 as long as the diameter of ocellus A. Chaeta s’ of ant.III in males present. Two prelabral chaetae. Outer maxillary lobe with three sublobal hairs and simple maxillary palp. Labium with three proximal and four basomedian chaetae, labial palp with reduced set of guards [A(1)B(3)C(0)D(3)E(4)] (as in Fig. 2). Postlabial chaetae 3+3. With 4–5 chaetae between medial line and pc3 on head. Frontal chaeta ap absent.

Chaetotaxy scarce (Figs 49, 50). Axial chaetotaxy 12–10,8/4,4,4,4. Macrochaetotaxy: 3(W),3(W)/2,2,3. Mdl macrochaetae in p-row on Th. II and III, sometimes weakly developed. Number of s-chaetae: 3,3/2,2,2,2,4 (s), 1,0/1,0,0 (ms) (Fig. 47). S-chaetae short, medial ones on Abd. I–III arranged behind Mdl macrochaetae. Sternite of Th. III without chaeta.

Legs as in *T.tardoki* sp. nov. Tibiotarsi I, II, III with 21, 21, 22 chaetae. Claw without teeth. Empodial appendage short, 0.2–0.3 as long as inner edge of claw. Ventral tube with 3+3 lateral and four posterior chaetae.

Retinaculum and furca absent. Retinacular field with 3–5 chaetae. Anterior furcal subcoxa with three (rarely four) chaetae, posterior one with four chaetae. Manubrial field with eight (rarely seven) chaetae (Fig. 48). Anal spines parallel, large, on high papillae. Papillae of inner pair sclerotised. Medial mesochaetae (a1) of Abd. V anterior to medial macrochaetae (a2) (Fig. 49). Arrangement of chaetae and spines on dorsum of Abd V as a2-a2/a1-a1 = 1.7–1.8; a2-a2/a2-eAS = 1.3–1.7. Males present.

#### Distribution.

**Figure 58.**
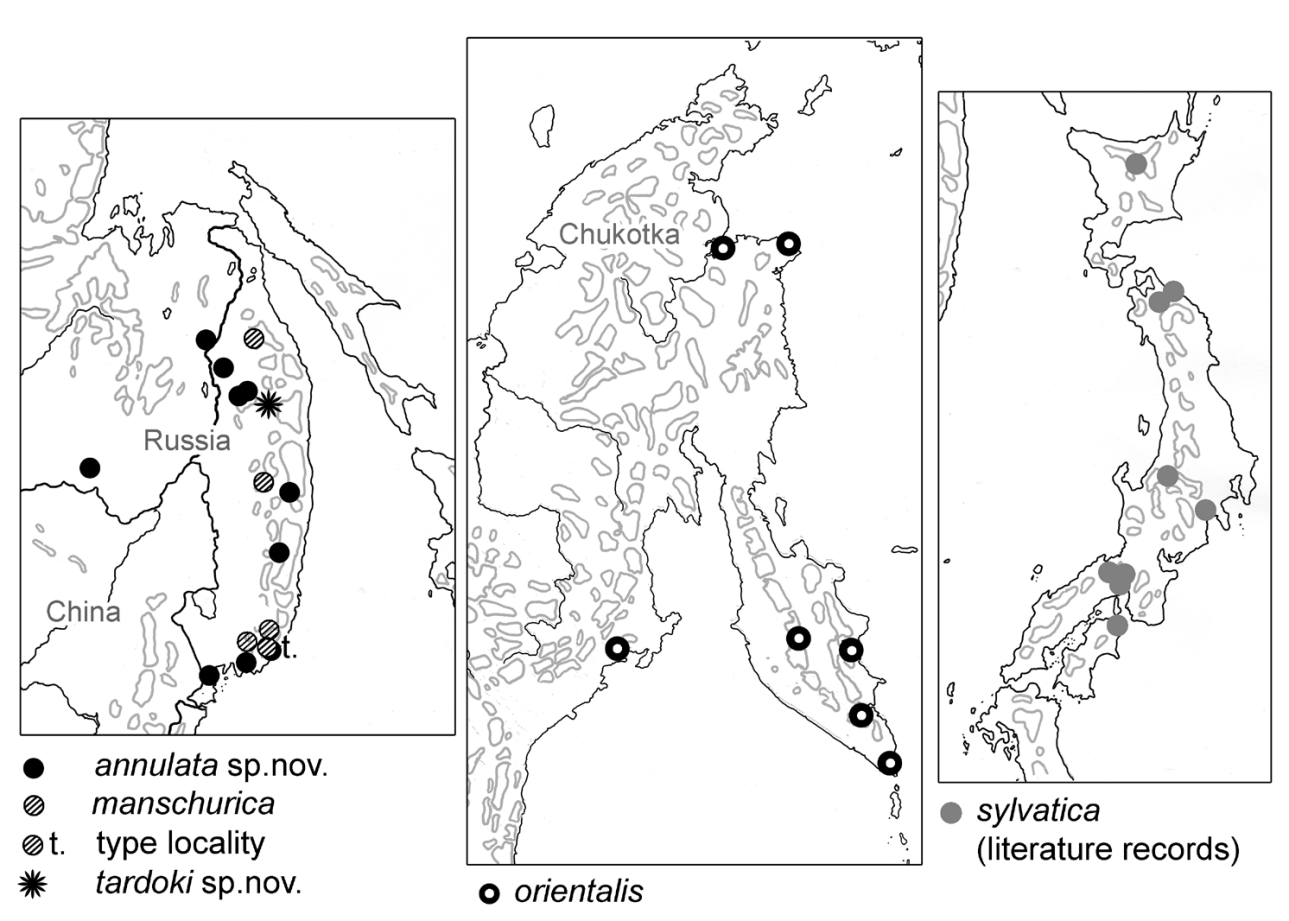
Records of five species of *Tetracanthella* in the Far East of Russia and Japan.

*Tetracanthellaorientalis* is widely distributed in northern part of the Far East of Russia (Fig. 58). In Magadanskaya region and Kamchatka it is the only known species of the genus. In Chukotka, *T.orientalis* can be recorded together with *T.sibirica* which has the similar appearance but belongs to another group of species.

#### Discussion.

See the remarks to *T.tardoki* sp. nov.

## Species of the ‘*wahlgreni*’ group

### Tetracanthella sibirica

Taxon classificationAnimaliaCollembolaIsotomidae

Deharveng, 1987

Tetracanthella arctica auct.
Tetracanthella cf. arctica auct.

#### Material from the Far East of Russia.

Chukotski AO: Anadyrski district, vicinities of Anadyr (holotype and paratype), leg. E. Bondarenko; ibidem, Anadyrski district, vicinities of Shakhterski, Volchikha River, leg. E. Bondarenko; ibidem, Iul’tinski district, Shmidta Cape, leg. K. Gorodkov; ibidem, Iul’tinski district, Wrangel Island, Somnitel’naya Bay, leg. K. Gorodkov.

#### Material from the Palearctic.

Yakutia, Bulunski Ulus, Bol’shoi Lyakhovski Isl. (Novosibirskiye Islands), mouth of Bol’shoi Etirikan River; ibidem, Bol’shoi Lyakhovski Isl., Shalourova Cape, leg. V. Bulavintsev.

#### Material from the Nearctic.

USA, Alaska, Kotzebue, 66.90°N, 162.59°W, 04.IX.1976, trough, moss & *Carex* litter, leg. R. Greenberg; Alaska, North Slope, 10 ml NW Franklin Bruffs, moss in active polygon, 70.26°N, 161.89°W, 17.VIII.1976, leg.A.F.; Alaska, North Slope, Icy Cape, trough between polygons, moss and *Carex* sp., 28.VIII.1976, leg. P. Connors; Alaska, North Slope, Canning River Delta, 70.05°N, 145.50°W, 23.VII.1980, several sites with *Dryas* sp., moss and *Carex* sp., leg. S. MacLean; Alaska, Norton Bay, Inglutalik River, moist tundra, leg. A.F.; Alaska, Nunivak Island, Duchikthluk Bay, 59.86°N, 166.07°W, 19.IX.1976 tundra with *Empetrum* sp., *Carex* sp., *Vaccinium* sp., lichens, leg. P. Michelson; Alaska, Point Barrow, 71.31°N, 156.66°W, 30.VIII.1976, thick moss, some algae and lichens; ibidem, moss , *Saxifraga* sp., *Cochlearia* sp. on sandy stream bank, leg. A.F.; Alaska, Cape Thompson, Ogoturuk Creek Basin, 68.16°N, 165.35°W, 11.VIII.1980, moss in tussock tundra, leg. D. & B. Murrey; Alaska, Chevak in Yukon, Kuskokwin Delta, 61.51°N, 165.26°W, 09.VII.1976, mesic upland, moss and *Betulanana*, leg. T. Seasted; Alaska, Cape Krusenstern, rather dry site, lichens, 67.25°N, 163.50°W, 03.IX.1976, *Vaccinium* sp., leg. R. Greenberg.

#### Distribution.

Common in Asiatic and American parts of Arctic. In the Far East of Russia was recorded from Chukotka, in Wrangel Island, Anadyrskij, Uil’tinskij, Chaunskij and Chukotskij districts, as *T.arctica* Cassagnau, 1959 or T.cf.arctica in older publications (Bondarenko 1975, Deharveng 1987, Babenko 2010, 2017, Martynova 1971, MacLean et al. 1978, and others). All Arctic records from the literature were summarized by Babenko and Fjellberg (2006).

#### Discussion.

The status of species is somewhat doubtful because morphological intergrading to *T.arctica* (Fjellberg 2007, 2015) which is distributed in Atlantic sector of Arctic. Specimens of *T.sibirica* from Asia fit to the first description (Deharveng 1987).

### Tetracanthella martynovae

Taxon classificationAnimaliaCollembolaIsotomidae

Potapov, 1997

#### Distribution.

The species is distributed in central part of Russian sector of Arctic. From the Far East it is known only in Chaunski district (Chaun Bay) of Chukotka, as “cf. britannica” by MacLean et al. (1978), which is the most eastern record of the species so far.

## Supplementary Material

XML Treatment for Tetracanthella annulata

XML Treatment for Tetracanthella sylvatica

XML Treatment for Tetracanthella manschurica

XML Treatment for Tetracanthella czernovae

XML Treatment for Tetracanthella tardoki

XML Treatment for Tetracanthella orientalis

XML Treatment for Tetracanthella sibirica

XML Treatment for Tetracanthella martynovae
